# Care My Way: Co‐Designing a Patient‐Held Resource to Improve Information Sharing Between Primary and Specialist Care for People With Cancer

**DOI:** 10.1111/hex.70498

**Published:** 2026-01-04

**Authors:** Reema Harrison, Bronwyn Newman, Ashfaq Chauhan, Kellie Holland, Clarel Philibert, Juliana Emerick, Kirsten Oataway, Elizabeth Manias, Carlene Wilson

**Affiliations:** ^1^ Australian Institute of Health Innovation Macquarie University Sydney New South Wales Australia; ^2^ Counterpart, Women's Health Victoria Melbourne Victoria Australia; ^3^ ONJ Centre, Austin Health Melbourne Victoria Australia; ^4^ Monash Nursing and Midwifery Monash University Melbourne Victoria Australia; ^5^ Centre for Epidemiology and Biostatistics, Melbourne School of Population and Global Health The University of Melbourne Melbourne Victoria Australia

**Keywords:** cancer, co‐design, communication, consumer engagement, cultural diversity, patient involvement, primary care

## Abstract

**Background:**

For many individuals, living with cancer means having a chronic and complex health condition that requires well‐coordinated care between primary and specialist settings. Continuity of care is often compromised by lack of shared information between providers about a person's circumstances, communication support needs, health information or cultural requirements. This project aimed to use co‐design with people from culturally and linguistically diverse backgrounds who have lived experience of cancer and their care providers to create a resource to enhance continuity of care when accessing multiple health providers.

**Methods:**

A co‐design group (*n* = 9) was formed comprising of people from different cultural and linguistic backgrounds with lived experience of cancer (*n* = 4) as survivors or carers, alongside healthcare providers (*n* = 5) working in primary care or as specialists. Co‐design group members were recruited from cancer support organisations, consumer organisations, primary health networks, hospital oncology services and professional networks nationally in Australia to provide diverse lived experience of cancer and professional expertise in the provision of cancer care. A series of four co‐design workshops were held, supported by asynchronous communication facilitated by an external agency to create and refine the final prototype resource.

**Results:**

The co‐design group created a person‐held tool called Care My Way for individuals with cancer to share socio‐cultural information with health providers. This resource was developed in response to an identified gap in person‐held resources that for people with cancer to communicate their socio‐cultural information beyond interpreter requirements, language spoken and country of birth so that health providers can understand how this may influence care. Care My Way comprises of four components through which people living with cancer can share information about (1) themselves, (2) their communication approach and support needs, (3) their cancer journey and care, and (4) their care requirements based on cultural and faith backgrounds. Using Care My Way seeks to promote relational and informational continuity of care between multiple health professionals for people with cancer whose care spans primary and specialist settings.

**Conclusions:**

Person‐held tools such as Care My Way provide an opportunity for people affected by cancer to identify critical information about themselves and their care, including the socio‐cultural information relevant to their care. Using Care My Way puts the individual at the heart of decision‐making about the information they wish to share and may provide a low‐cost, accessible and flexible method for individuals to share information about themselves, their culture and their health between primary and specialist settings in a bid to improve quality of care. Evaluative studies are now required to determine the effectiveness of this tool for improving continuity of care.

**Patient or Public Contribution:**

This study is part of a 4‐year project that was designed with consumers who have lived experiences of cancer as a patient or carer from diverse cultural and linguistic backgrounds in Australia. The project included consumer investigators and a Consumer Advisory Group throughout the lifecycle. The co‐authors of this manuscript include consumers (C.P., J.E.) who were members of the co‐design group who wished to contribute as authors.

## 1 Introduction

For many individuals, living with cancer means living with a chronic and complex health condition that requires well‐coordinated care over extended periods of time [1]. Health system models for high‐quality chronic disease management highlight the importance of effective collaborative care between primary and specialist settings to enable person‐centred care [2]. Collaborative care between a specialist‐led clinical team and a primary care provider is important to ensure that personal and health information is shared so that care best meets an individual's needs [2]. For those with cancer, collaborative care models are often described as shared care, involving a specialist oncology team and primary care provider [3]. In an effective shared care model, individuals should experience enhanced continuity of care (CoC) and smoother transition between specialist and primary care services when required.

CoC describes coordinated, timely and coherent delivery of health services. CoC is multidimensional, characterised by three domains: relational continuity (promoting the relationship between patients and care provider/s); management continuity (promoting coordination across a multidisciplinary care team that spans multiple service providers); and informational continuity (supporting the exchange of relevant clinical and personal information). The availability of patient health and social information across the care continuum is identified as a key enabler; all providers require up‐to‐date information about a person's condition, history and care plan [4].

Information about an individual's health condition, history, care plan and personal context including their support needs is particularly important for people from culturally and linguistically diverse (CALD) backgrounds to ensure that their care is responsive to their needs and preferences. The term CALD is used in Australia throughout health system policy and practice to describe individuals born outside Australia, who have one or more parents born outside Australia, speak a language other than English at home or require an interpreter for their communication [5]. Disparities in cancer care among people from CALD backgrounds are notable, spanning from lower cancer screening, less timely diagnosis, to greater likelihood of poor outcomes in specialist care settings [6, 7]. There are multifactorial contributors to poorer care quality and outcomes, but substantial evidence indicating that the lack of shared information between health providers is a key contributor [8]. When information about language support needs, migration‐related factors (such as trauma, past healthcare received, visa status), religious and faith‐based requirements is not accessible to providers, individuals are required to repeat information or risk having their needs unmet.

Whilst case managers and case conferences have been used to promote CoC [3], cancer survivors have highlighted the need for person‐held information to support CoC throughout their health care. A person affected by cancer is the central point of connection between their healthcare providers; they can verify information, provide additional information about themselves or their care and identify mistakes in care. This central role is recognised internationally in healthcare quality improvement [9, 10]. For people from CALD backgrounds specifically, person‐held information provides an opportunity for individuals to share the information about themselves that they wish to and that is considered relevant so that person‐centric care is achieved [4, 11].

A variety of patient‐held records, logbooks and care plans have been produced specifically aiming to promote CoC for people with cancer [11]. Whilst these have been identified as useful to promote sharing of health information, which may promote management CoC, there has been a less focus on the sharing of personal and social information that may promote relational CoC; an enduring therapeutic relationship between a patient and specific healthcare provider [11, 12]. Using a person‐held resource that includes information about personal and social context may be particularly important in the care of cancer survivors from CALD backgrounds who have highlighted the need for information about their communication preferences, cultural and faith‐based requirements and personal support needs to be understood to ensure that they can access quality care [13]. At present, person‐held tools focus on clinical history and survivorship needs, with socio‐cultural data limited to language spoken, country of birth, religion and interpreter needs [14]. In the disability sector, such tools have incorporated patient‐identified communication needs and preferences, but socio‐cultural data have not been a central feature [15]. Whilst these data may inform care, these closed response items do not provide a person‐centric approach for individuals affected by cancer to identify the socio‐cultural considerations that matter to them. Health providers who only receive information about interpreter needs, country of birth, language spoken at home and religion may not have the information they need to apply this knowledge in action during care beyond ensuring interpreters are available.

Use of co‐design has been increasingly documented in primary and integrated care as a method to design and develop person‐centric quality improvement initiatives at local practice level but also to ignite system‐wide change [16]. Situated within communities, primary care settings have been leveraged for community‐based co‐design to identify and respond to local population needs [17, 18]. Conducted as part of a 4 year national programme of multicultural health research in relation to cancer care among diverse cultural and linguistic communities in Australia, this study sought to use co‐design to create a resource to improve CoC for and with people affected by cancer that meets the need of individuals from a range of cultural and linguistic backgrounds. Rather than developing targeted resources for one or more cultural, linguistic or ethnic groups, the national research project sought to enhance health service responsiveness to the multicultural Australian community.

## 2 Method

Co‐design *‘brings together lived experience, lived expertise and professional experience to learn from each other and make things better by design* [19]*’.* In the current project, co‐design was employed to promote collaborative dialogue between people from a range of cultural and linguistic backgrounds with lived experience of having or supporting someone with cancer and their care providers. Currently, there is no preferred reporting guideline for co‐design projects. Consequently, we used the PRODUCES framework (PRoblem, Objective, Design, (end)‐Users, Co‐creators, Evaluation and Scalability) to guide the reporting of this study [20]. This framework was developed as a set of principles and recommendations for the reporting of co‐creation in public health.

Ethics: This study received ethics approval from National Health and Medical Research Council (NHMRC) accredited Western Sydney Local Health District Human Research Ethics Committee: Reference number (2020/ETH0965) and (2021/ETH00532).

Objective: The objective of this project was to identify and develop a resource to enhance CoC through co‐design between people from CALD with lived experience of having or supporting someone with cancer and their care providers. As a result of the co‐design process, the co‐design group determined that the resource would be a person‐held tool because such tools have been used to promote CoC in a range of health settings. Specifically, the tool would seek to address a gap in existing person‐held tools for people with cancer. Existing tools provide limited opportunities for people with cancer to share the socio‐cultural information that matters to them. Given the inter‐ and intra‐cultural differences throughout CALD communities, the resource sought to facilitate conversation about a diversity of individual needs and preferences in communicating with multiple care providers.

Co‐design planning: Drawing from our 4‐year programme of research, which encompassed a series of co‐design projects with CALD communities, we adopted a range of strategies to optimise inclusion when planning our co‐design [21, 22]. Strategies included the use of a multicultural consumer network to promote the research and address queries ahead of participating, identifying cultural and linguistic support needs of co‐design members ahead of the process, the inclusion of bicultural facilitators with lived experience of cancer care during co‐design workshops. The term consumer is adopted throughout this manuscript to describe of people with lived experience of being a patient, family member or carer for someone with cancer. This term is used at federal and state level throughout the Australian health system to describe patients, families and the general public based on the preferences expressed by people who use the Australian health system [23]. A range of wider strategies for good co‐design practice were further adopted to promote inclusion and engagement of diverse members throughout process [21], including addressing communication support needs, providing materials ahead of time, ensuring suitable meeting timing and location and providing remuneration for involvement.

### 2.1 Co‐Design Group Members

The co‐design group was formed to comprise people with lived and professional experience relevant to CoC in cancer. Nine members were recruited; four people who contributed lived experience of cancer as a survivor (*n* = 2) or as a carer (*n* = 2) from different cultural and linguistic backgrounds. Five further members included three primary care professionals and two specialist cancer care professionals who contributed professional expertise in the challenges of CoC and considerations when working with CALD communities. Members were recruited from cancer support organisations, cancer services who were partner organisations in the research programme and consumer organisations via social media and email advertisements. In the final workshop, the co‐design group was supported by five clinical‐academic staff from four different cultural backgrounds who provided professional expertise in embedding theory into co‐design as part of the synchronous and asynchronous prototyping process. In bringing professional expertise in multicultural health, CoC, psychology, nursing, pharmacy, psycho‐oncology, allied health and social work, these individuals supported the consumer and clinician team to build the digital tool by providing theoretical and practical advice relevant to the goals of the group [24, 25].

**Figure 1:**
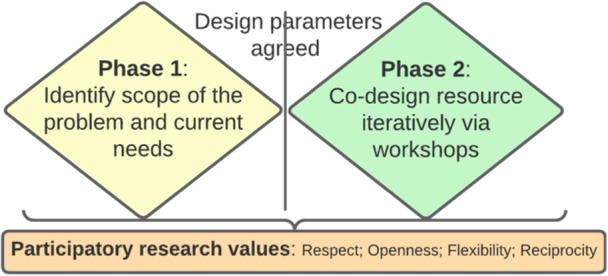
Co‐design double diamond approach.

Co‐design procedure: Once group members were recruited, a co‐design group was convened and the support needs of members to engage in the process were determined and addressed ahead of commencement. Co‐design members (*n* = 9) developed and agreed upon the terms of reference for the group in their initial meeting, with behaviour ultimately guided by an agreed code of care that was designed to support equitable participation. The group was facilitated through four in person and online workshops, telephone conversations and emails over 12 weeks. At the outset of each workshop, members were reminded of the co‐design code of care, and the aim and purpose were agreed. Three workshops were conducted with an experienced facilitator with a background in social work and a bicultural facilitator with lived experience as a carer for parents and a sibling with cancer. The final workshop was facilitated by an external facilitator from a digital design agency to create a digital prototype. Design took place using a double diamond approach (Figure 1) comprising of two phases [26]. In the first phase, lived experiences and existing literature were drawn upon to scope the problem and design parameters. In the second phase, solutions were proposed and a resource created.

### 2.2 Phase 1: Scoping the Problem Through Contextual and Experiential Data

Multi‐source data were gathered over 24 months about the experiences of people from CALD backgrounds with cancer and the quality of their care. Data comprised a medical record review conducted with > 600 patient records across four cancer services in the Australian states of New South Wales and Victoria, systematic review of 27 patient engagement strategies to improve healthcare quality [27], a document analysis of consumer engagement frameworks [28], a national stakeholder analysis of the use of patient engagement methods with CALD communities [29], qualitative interviews conducted with 54 cancer care staff [30] and 19 consumers [31] about their experiences of cancer care and observational data about engagement opportunities during cancer care [32]. These data were examined by co‐design members in the first workshop. Members determined the key problems that were important and feasible to address through discussion and voting. Drawing upon these data, their lived experience and expertise, the co‐design group determined that to improve CoC between primary and specialist care for people with cancer from CALD backgrounds, a flexible, digital person‐held resource was required and became the aim of the co‐design process. We sought to develop a tool between patients, carers and health professionals that was not culturally targeted or tailored, but offered a mechanism that enables people to document cultural and linguistic factors that are important to them and how these matters influence their care for use by consumers and care providers.

### 2.3 Phase 2: Developing a Solution

**Figure 2:**
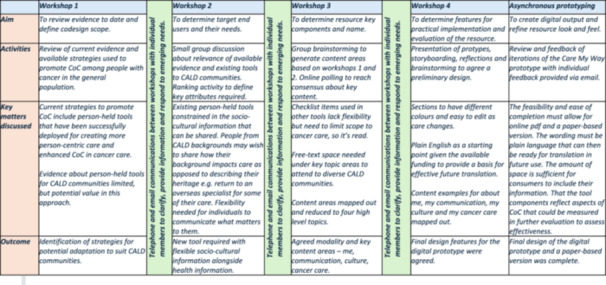
Co‐design workshop activities and outcomes.

**Figure 3:**
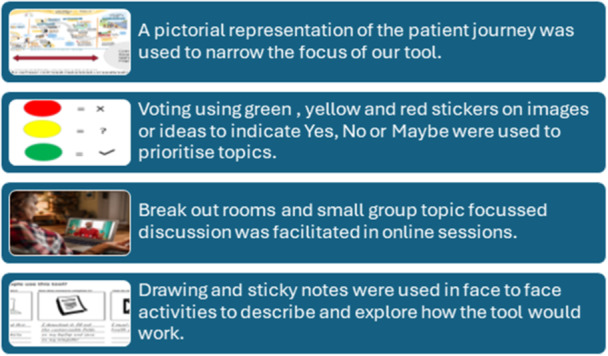
Examples of co‐design activities.

Three further workshops were conducted to design the tool (Figure 2). In the second workshop, consumer and provider co‐design members explored currently available resources and identified a person‐held resource along with the key content that should be captured. The third workshop focused on developing the look and feel of the resource and naming this ‘Care My Way’. During these workshops, techniques including brainstorming, polling, scenario‐based activities and small group discussions (Figure 3) were used to design the elements of the resource. Key decisions made at each point are summarised in Figure 2.

Following the initial prototype design, a fourth workshop was convened in which the project team engaged a digital designer to provide external facilitation, create and refine further iterations of the Care My Way resource. To provide practical and theoretical advice about the resource, five multidisciplinary academics from the wider 4‐year research programme contributed to the workshop and asynchronous prototyping. Specifically, these individuals were able to provide insights from evidence in patient‐held resources in cancer, used for quality improvement, best practice approaches to developing multicultural health resources, existing theory and clinical perspectives from a broader lens that may aid implementation of the resource in the Australian health system. Following the fourth workshop, iterative asynchronous feedback was provided by co‐design group members and multidisciplinary academics on the prototype via email until a final version was mutually agreed.

### 2.4 Output: Care My Way Resource

**Figure 4:**
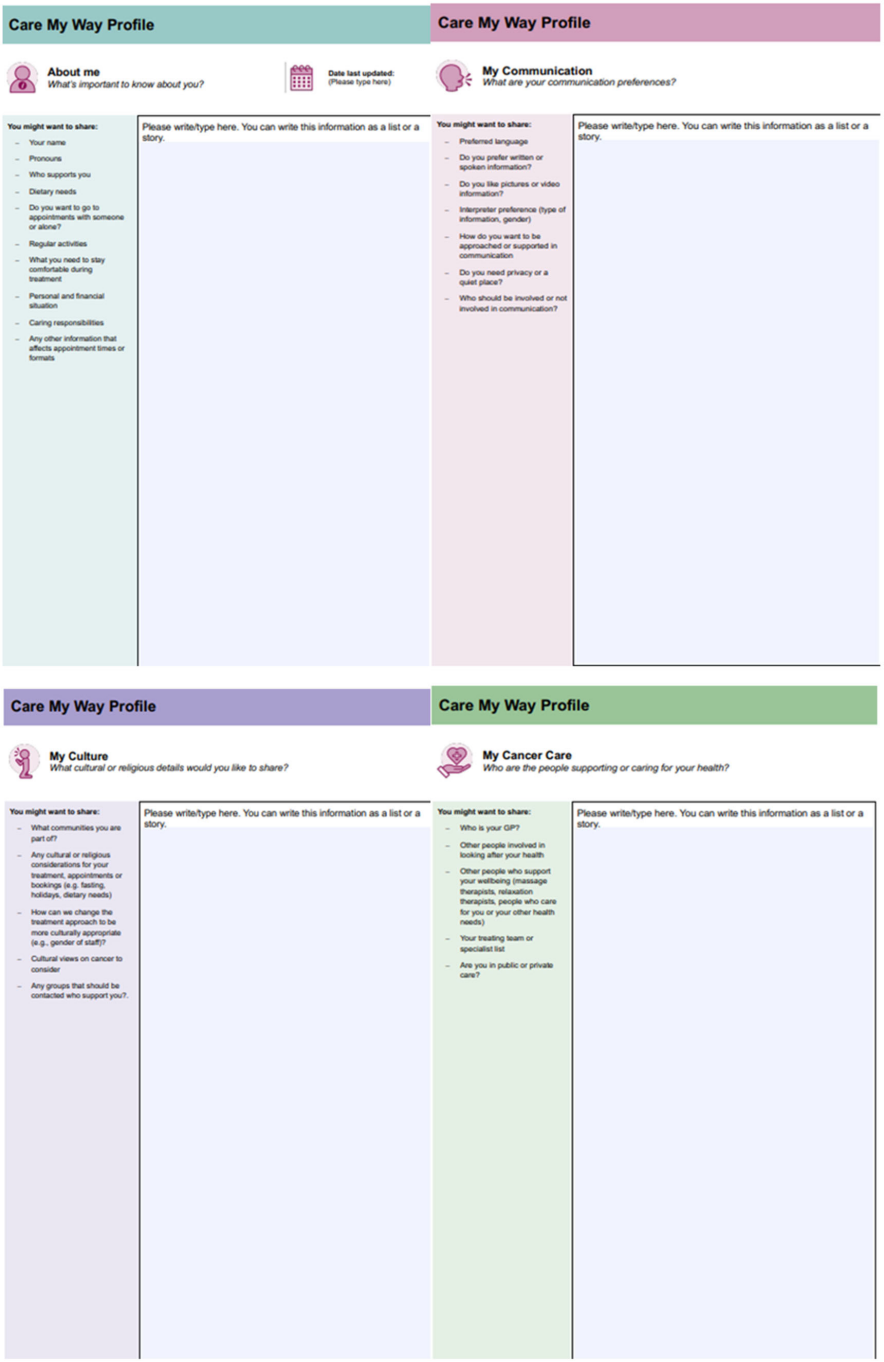
Care My Way resource.

Care My Way was designed to provide an opportunity for people affected by cancer to share personal information with their healthcare team. Because people with cancer are the expert in their own needs, Care My Way provides a mechanism to manage and control the information they wish to share with others. Co‐design members discussed the evidence demonstrating that cultural background, ethnicity, religion, faith and/or language can have a range of implications for their cancer care. Individuals may choose to share a variety of personal and social information; the nature of which may differ from one individual to another. In designing Care My Way, the resource sought to provide an opportunity for individuals to choose what they wish to share with care providers. As a result of the co‐design activities, Care My Way was not designed for a specific cultural group or specifically for people who have low English proficiency. However, the resource is in the process of being made available in further languages alongside the plain English text for use by the consumer and health organisations. At this stage, Care My Way can be used as a flexible, person‐held digital (or printed) resource by people from a diverse range of backgrounds who wish to share information about themselves, their health, their culture and backgrounds, their communication and the resulting implications for care. A central purpose of Care My Way is to reduce the need to repeat personal, cultural and health information to multiple health professionals while accessing primary and specialist care from multiple providers. Care My Way comprises four sections (Figure 4): (1) About me, (2) my communication, (3) my culture and (4) my cancer care.

### 2.5 Evaluation and Scalability

Data on co‐design members’ experiences of the co‐design process and their satisfaction with it were gathered throughout the CanEngage Project, with an initial report about experiences of CALD co‐facilitators prepared [33] and an outcome evaluation in process. Care My Way is now freely available (Care_My_Way_For_Patients_v2.pdf) for consumers and healthcare providers to use. The next step of this study is to evaluate the feasibility and acceptability of Care My Way, ahead of testing its effectiveness with a representative sample. We intend to collaborate with cancer support organisations involved in the co‐design to conduct a feasibility and acceptability study evaluating the use of the tool to inform a larger scale effectiveness trial to determine impacts of Care My Way on CoC for people from CALD backgrounds with cancer and their care providers.

## 3 Discussion

Consumers identified a need for person‐held resources to support the effective sharing of health, personal and social information with health care providers when navigating cancer. By engaging with people affected by cancer who come from a range of CALD backgrounds and their healthcare teams, this study revealed that a person‐held tool must be flexible and responsive to individual circumstances and preferences. By making available a resource that can be populated by the patient, including information they consider important, Care My Way seeks to facilitate individuals to share the information that is relevant to their care at any given point in the journey. Specifically, consistent understanding between health providers participating in shared care about a patient's background, values and needs may be minimised when they all have access to this type of patient‐derived documentation.

Care My Way compliments a range of person‐held tools that have sought to improve care continuity, quality and safety [11, 27]. Examples include patient‐held records that intend to improve communication and information exchange between and within different levels of care [11] along with bedside tools for use during healthcare encounters [27], and alert cards to flag at risk patients [34]. Whilst patients, families and health professionals acknowledge the potential value of such tools, challenges in ensuring their suitability for different patient groups and agreement about the function of such tools continue to present barriers to their use. Implementation studies have identified that despite the value of tools to promote patient engagement in care, barriers due to inconsistent use, tools not being embedded in workflows and variable support for their use in clinical settings may inhibit their successful use [35]. Greater understanding of the implementation factors that support the effective use of person‐held tools is required to realise their potential impacts.

Our work further provides insight in the use of co‐design for quality improvement in primary and integrated care. Our experiences in developing Care My Way in the Australian health system reflect international evidence that suggests co‐design in primary care can inform and ignite health equity initiatives driven by local needs [17]. When applied in a primary care setting, co‐design sessions may provide an opportunity for different members of the healthcare team and consumers from a range of different backgrounds to connect when they might not otherwise be able to do so to identify problems and develop solutions. The inclusion of members from a range of care and support services was vital in the primary and integrated care context to provide consideration of how the tool might be used between settings, and constraints or opportunities in diverse service delivery contexts.

Yet bringing together individuals from a range of different backgrounds and circumstances was challenging. Practical challenges for conducting primary care co‐design and associated mitigation strategies were therefore identified. Co‐design members were distributed across a large geographic area, with varying personal and professional circumstances. Our co‐design therefore took place on a continuum from entirely in person to entirely asynchronous [36]. The first and final co‐design workshops were held in‐person with the remainder held partially or fully online. Taking a hybrid approach created space for community collaboration [37] yet offered a flexible and accessible approach. Asynchronous prototyping aided the provision of detailed feedback over an extended duration from all users bringing diverse perspectives. Whilst individuals were supported through this hybrid approach, extensive planning was required along with communication between sessions to support contributions. It was vital to consider the time, remuneration, communication skills and documentation required to enable effective engagement in this dynamic co‐design approach.

## 4 Conclusion

Using co‐design we produced a person‐held tool to provide an opportunity for people from a range of cultural and linguistic backgrounds affected by cancer to provide critical information about themselves and their care. The Care My Way tool positions the person at the centre of their care, and may provide a low‐cost, accessible and flexible method for people to share information about themselves, their culture and their health between primary and specialist settings in a bid to improve their quality of care. This tool aims to provide greater visibility of a person's cultural, religious, and community needs, alongside their health information toward a more tailored approach to care. By recognising the broader social and personal factors that influence care, Care My Way seeks to enhance communication, engagement and patient experience. Evaluative studies are now required to determine the effectiveness of Care My Way for improving CoC.

## Author Contributions

**Reema Harrison:** conceptualisation, formal analysis, writing – original draft, writing – review and editing, supervision, methodology, investigation, funding acquisition, resources. **Bronwyn Newman:** conceptualisation, data curation, investigation, methodology, writing – review and editing, formal analysis, supervision. **Ashfaq Chauhan:** writing – review and editing, formal analysis, data curation, conceptualisation, methodology, investigation. **Kellie Holland:** conceptualisation, writing – review and editing, formal analysis. **Clarel Philibert:** formal analysis, writing –review and editing, conceptualisation. **Juliana Emerick:** conceptualisation, formal analysis, writing – review and editing. **Kirsten Oataway:** conceptualisation, formal analysis, writing – review and editing. **Elizabeth Manias:** conceptualisation, data curation, writing – review and editing, methodology, investigation, funding acquisition, supervision. **Carlene Wilson:** conceptualisation, data curation, methodology, investigation, supervision, writing – review and editing, funding acquisition.

## Ethics Statement

This study received ethics approval from National Health and Medical Research Council (NHMRC) accredited Western Sydney Local Health District Human Research Ethics Committee: Reference number (2020/ETH0965) and (2021/ETH00532).

## Consent

Written consent was sought from all participants.

## Conflicts of Interest

The authors declare no conflicts of interest.

## Data Availability

Research data are restricted to the research team identified in the ethical approval.
